# Using animal models for the studies of schizophrenia and depression: The value of translational models for treatment and prevention

**DOI:** 10.3389/fnbeh.2022.935320

**Published:** 2022-08-24

**Authors:** Daniela L. Uliana, Xiyu Zhu, Felipe V. Gomes, Anthony A. Grace

**Affiliations:** ^1^Department of Neuroscience, University of Pittsburgh, Pittsburgh, PA, United States; ^2^Department of Psychiatry, University of Pittsburgh, Pittsburgh, PA, United States; ^3^Department of Psychology, University of Pittsburgh, Pittsburgh, PA, United States; ^4^Department of Psychiatry and Behavioral Sciences, University of California, San Francisco, San Francisco, CA, United States; ^5^Department of Pharmacology, Ribeirão Preto Medical School, University of São Paulo, Ribeirão Preto, SP, Brazil

**Keywords:** schizophrenia, depression, hippocampus, plasticity, prefrontal cortex, dopamine

## Abstract

Animal models of psychiatric disorders have been highly effective in advancing the field, identifying circuits related to pathophysiology, and identifying novel therapeutic targets. In this review, we show how animal models, particularly those based on development, have provided essential information regarding circuits involved in disorders, disease progression, and novel targets for intervention and potentially prevention. Nonetheless, in recent years there has been a pushback, largely driven by the US National Institute of Mental Health (NIMH), to shift away from animal models and instead focus on circuits in normal subjects. This has been driven primarily from a lack of discovery of new effective therapeutic targets, and the failure of targets based on preclinical research to show efficacy. We discuss why animal models of complex disorders, when strongly cross-validated by clinical research, are essential to understand disease etiology as well as pathophysiology, and direct new drug discovery. Issues related to shortcomings in clinical trial design that confound translation from animal models as well as the failure to take patient pharmacological history into account are proposed to be a source of the failure of what are likely effective compounds from showing promise in clinical trials.

## Background

Animal models have been highly effective in advancing our understanding of psychiatric disorders in terms of pathophysiology, disease development, symptomatology, and in proposing novel targets for therapeutic intervention. However, it is also clear that animal models cannot replicate in a precise manner the complex syndrome present in humans. For this reason, animal models must be carefully selected based on parallels to human conditions and circuit-specific alterations that can lead to pathophysiology. In this sense, it is proposed that, to be meaningful, animal models should (1) possess similarity in symptoms, such that the observed abnormalities of the model have clinical correlates in patients (face validity); (2) replicate the neurobiological bases of the human condition (construct validity); and (3) predict responsiveness to drugs currently used to treat the disorder (predictive validity) (Howland et al., 2019).

The initial animal models to study mental disorders were based on lesions or acute drug administration. Such studies have been influential in the early days of investigation of disease states. For example, it was the studies of amphetamine and other dopaminergic agents that gave us insight into the role of dopamine in schizophrenia (Smythies et al., 1967; Snyder, 1973). This was further substantiated by studies showing that antipsychotic drugs were primarily dopamine antagonists (Creese et al., 1976; Seeman et al., 1976). However, such acute models often failed to replicate the clinical conditions. Therefore, whereas baseline levels of dopamine in schizophrenia are not substantially different from controls (Abi-Dargham et al., 2000), amphetamine induces a 20–30-fold increase in dopamine turnover (Joel et al., 1997). Furthermore, although lesion studies, such as prefrontal cortical lesions, can replicate some executive function deficits found in schizophrenia (Joel et al., 1997), it is also apparent that patients do not have a “hole” in their prefrontal cortex.

There are also issues related to the types of behavioral tests to evaluate the models. Amphetamine-induced hyperlocomotion has been advanced as a means to correlate the hyperdopaminergic state present in schizophrenia. However, recent studies have shown that this is not an accurate model (van den Buuse, 2010). Studies in humans have shown that the psychosis-relevant hyper-responsivity of the dopamine system is located primarily in the associative striatum (Kegeles et al., 2010; McCutcheon et al., 2018). However, amphetamine-induced hyperlocomotion is instead shown to be a hyperfunction of dopamine transmission in the ventromedial striatum (Kelly et al., 1975), which is involved more in affective processes than in salience/psychosis (Floresco, 2015). In addition, acute amphetamine does not induce changes related to the negative symptoms and cognitive deficits seen in patients with schizophrenia (Krystal et al., 2005). Another example would be the studies used to assess related features of depression. Thus, the forced swim test has been long used in evaluating antidepressant medications; however, this has come under scrutiny with regard to its specificity for a negative affective state and despair related to the immobility measured in the test. Recently, it was associated with stress coping and learning processes which antidepressants would increase active coping over passive coping but do not necessarily reflect the negative affect (Molendijk and de Kloet, 2015; Commons et al., 2017; Armario, 2021). The same can be extended to sucrose preference; although it has been used as a standard in depression research, the typical deficits seen are very small in magnitude (Willner et al., 1987; He et al., 2020). Further, it can be argued that deciding between two options one of which is rewarding is not an effective means to assess anhedonia; a better approach would be to see if the animal will expend effort to reach a desired reward, which should be more translatable. Therefore, using validated behavioral models for assessing animal models is fraught with problems.

Another approach would be to utilize comparisons, both with respect to model generation and evaluation, that are based on more readily translatable measures. This is consistent with the NIMH’s *Research Domain Criteria* (RDoC) framework for investigating mental illnesses, which has been inspiring novel animal modeling strategies to conceptually deviate from a deterministic principle and to incorporate clinically relevant and objectively measurable endophenotypes. Thus, given that most disorders show genetic association, it is assumed that the pathology is present from birth, and represents a more developmental alteration. Below we describe examples based on this reasoning applied to research using animal models for the study of schizophrenia and depression.

## Making a case for the use of animal models in schizophrenia research

Schizophrenia often emerges in late adolescence/early adulthood and is characterized by a variety of symptoms classically divided into three groups: positive, negative, and cognitive symptoms (Kahn et al., 2015). Positive symptoms include hallucinations, delusions, and thought disorders. Negative symptoms include blunted affect, alogia, anhedonia, social withdrawal, and avolition. Cognitive dysfunction involves deficits in working memory, attention, and processing speed, and trouble focusing. Schizophrenia has multifactorial etiology that includes genetic risk and exposure to socioenvironmental adversities (Miller et al., 2001; Henriksen et al., 2017; Popovic et al., 2019; Berthelot et al., 2022). Animal models for the study of schizophrenia comprehensively use genetic models, prenatal interventions, pharmacological models, and stress-based protocols during prepubertal periods to recapitulate the disorder features (Lipska and Weinberger, 2000; Meyer and Feldon, 2010; Modinos et al., 2015; Gomes et al., 2019).

Weinberger and colleagues used information regarding smaller hippocampal volumes in patients with schizophrenia (Weinberger, 1987) to induce an early-life hippocampal lesion in rats (neonatal ventral hippocampus lesion—NVHL—model), which upon examination as adults showed states consistent with schizophrenia (Lipska et al., 1993). In the NVHL model, lesion of the ventral hippocampus at PD7 induces behavioral alterations later in life consistent with schizophrenia, such as increased responsivity to psychostimulants and stress, decreased sociability, and impairments in cognitive function and prepulse inhibition (Lipska et al., 1993; Chambers et al., 1996; Becker et al., 1999; Grecksch et al., 1999; Lipska et al., 2002; Tseng et al., 2009). However, the lesion extent can impact the outcomes in which a small lesion is more effective to recapitulate hippocampal hyperexcitability (Patricio O’Donnell, personal communication) and increased dopamine responsivity (Swerdlow et al., 2001).

While a hippocampal lesion may not temporally replicate the pathology seen in humans and does not take genetic predisposition into account, it did provide insights into novel developmental changes. To explore preexisting neurodevelopmental factors in the pathophysiology of schizophrenia, we have used a developmental disruption model based on the administration of the DNA alkylating agent methylazoxymethanol acetate (MAM) to pregnant rats at gestational day 17. This date was chosen since it is functionally equivalent to the human second trimester, during which exposure to severe influenza infections can confer susceptibility of the offspring to schizophrenia (Barr et al., 1990). The result was that rats exposed to MAM in gestation showed characteristics as adults consistent with schizophrenia, including deficits in executive function behaviors, impaired latent inhibition and social interactions, and hyper-responsivity to amphetamine and the NMDA receptor antagonist phencyclidine (PCP) (Lodge and Grace, 2009). But importantly, the correspondence extended beyond behavior, showing anatomical changes (cortical thinning due to loss of neuropil and loss of parvalbumin-containing GABAergic interneurons), limbic hippocampal hyperactivity, and increased dopamine neuron population activity (Moore et al., 2006; Lodge and Grace, 2007; Lodge et al., 2009), all consistent with what has been reported in patients with schizophrenia (Modinos et al., 2015). In addition, similar to the human condition, most of the schizophrenia-like changes in the MAM model appear in late adolescence/early adulthood (Gomes et al., 2016).

An alternate approach was to utilize known risk factors in schizophrenia. Thus, it has been shown that severe influenza infections during gestation can increase the incidence of schizophrenia births (Barr et al., 1990). To approximate this in rodents, studies were done to model maternal immune activation, mainly through the Poly I:C gestational injection. Poly I:C is a synthetic double-stranded RNA, which approximates the conditions present with severe viral infections (Meyer and Feldon, 2012). In rodents, offspring of pregnant rats treated with Poly I:C express behavioral dysfunctions associated with schizophrenia (Wolff et al., 2011; Meehan et al., 2017), such as deficits in working memory and sensorimotor gating, and increased sensitivity to amphetamine, which can be alleviated with antipsychotic drug treatment (Knuesel et al., 2014; Reisinger et al., 2015). Neurobiological features are also presented, including reduced cortical thickness and hippocampal volume, and aberrant dopaminergic function (Knuesel et al., 2014; Meyer, 2014).

Genetics constitutes another domain of crucial risk factors for schizophrenia. Historically, genetic research on schizophrenia emphasized the search for “candidate gene(s)” in case-control studies. To date, over 1,000 genes have been tested but the overall results have been disappointing (Gejman et al., 2011; Mitchell et al., 2011; Henriksen et al., 2017), mainly due to issues of non-replication and/or lacking statistical power. Nonetheless, some of the most cited candidate genes have been targeted to generate genetic model animals, including *disrupted-in-schizophrenia 1* (DISC1), *neuregulin-1* (NRG1) and its receptor *ERBB4*, and *dystobrevin-binding protein 1* (DTNBP1) (Johnstone et al., 2011; Jones et al., 2011). Although targeted genetic engineering (often deletion) of those “candidate genes” tend to phenotypically recapitulate behavioral alterations in schizophrenia to various extents, recent evidence from more advanced molecular genetic studies suggest that the genetic architecture in schizophrenia may involve only a little contribution from highly penetrant mutations (Henriksen et al., 2017). Indeed, recent genome-wide association studies support that the genetic risk in schizophrenia is conferred by a large number of common alleles (Schizophrenia Working Group of the Psychiatric Genomics Consortium, 2014; Trubetskoy et al., 2022), which is extremely difficult to model in research animals due to difficulties to control allelic specificity and dosage (Mitchell et al., 2011).

Another prominent risk factor in schizophrenia is stress (van Winkel et al., 2008). Therefore, even in individuals without a family history of psychosis, childhood trauma is associated with a substantial increase in risk for schizophrenia (Popovic et al., 2019). Furthermore, in studies of children at risk for schizophrenia based on family history, Eve Johnstone and colleagues found that those children that were hyper-responsive to stress were more likely to transition to schizophrenia later in life (Miller et al., 2001). It was also found that clinical high-risk individuals have increased sensitivity to stress-induced dopamine release as same antipsychotic-naïve patients with schizophrenia which show antecedents of dopaminergic sensitivity to stress (Mizrahi et al., 2012). Drawing from these observations, we found that in the MAM model examined prepubertally, which is before the onset of the hyperdopaminergic state, the rats showed increased anxiety in the elevated plus maze, increased stress responsivity shown by heightened stress-induced ultrasonic vocalizations (Du and Grace, 2013; Zimmerman et al., 2013), and the basolateral amygdala (BLA), an area associated with stress and anxiety (Daviu et al., 2019), was firing at a constant high level (Du and Grace, 2016). Furthermore, if anxiety was alleviated in the MAM rats prepubertally either by administering diazepam, the pathology in the adult was circumvented (Du and Grace, 2013, 2016). Such insights provided a strong link between the human literature on early trauma and the MAM model. An increased responsivity to stress has also been described in the maternal immune activation and NVHL models (Lipska et al., 1993; Giovanoli et al., 2013).

## Making a case for the use of animal models in depression research

Major depression represents the most prevalent psychiatric condition worldwide in which patients express symptoms, such as depressed mood, anhedonia, fatigue, weight changes, and suicidal idealization (American Psychiatric Association, 2013; Otte et al., 2016). Adverse environmental factors, genetic background, and their interaction are the main risk factors for depression (Cross-Disorder Group of the Psychiatric Genomics Consortium, 2013; Richter-Levin and Xu, 2018; Silva et al., 2021). Animal models for depression often involve stress exposure and evaluate anhedonia, helplessness, motivation, and despair behaviors, as behavioral features; some examples are the chronic mild stress (CMS) model, learned helplessness (LH), and maternal separation (Schmidt et al., 2011; Czéh et al., 2016; Maier and Seligman, 2016; Antoniuk et al., 2019; Kaufling, 2019; Su and Si, 2022).

Depression has been linked to hyperactivity of the subgenual anterior cingulate cortex (sgACC) (Drevets et al., 2008b; Pizzagalli and Roberts, 2022), which is analogous to the infralimbic portion of the prefrontal cortex (ilPFC) in rodents (Laubach et al., 2018). All effective treatments were found to attenuate this sgACC hyperactivity (Drevets et al., 2008b), including local deep brain stimulation which is an effective intervention in treatment-resistant depression (Mayberg et al., 2005). Mimicking the human condition, animal models relevant for depression show increased ilPFC activity (Drevets et al., 2008b; Moreines et al., 2017b).

Preclinical studies have contributed to creating a strong base of circuit dysfunction, behavioral dysregulation, and antidepressants mechanism. For example, CMS commonly causes rodents to show changes related to anhedonia, amotivational behaviors, and decreased dopamine system activity (Willner et al., 1987; Belujon and Grace, 2017; Antoniuk et al., 2019). In addition, either direct ilPFC activation in normal rats or in rats exposed to CMS express this hypodopaminergic state (Patton et al., 2013; Chang and Grace, 2014) and ilPFC inactivation restores the normative dopamine activity in rats subjected to CMS (Moreines et al., 2017b), suggesting a relevant translational circuit dysfunction of the human condition.

**FIGURE 1:**
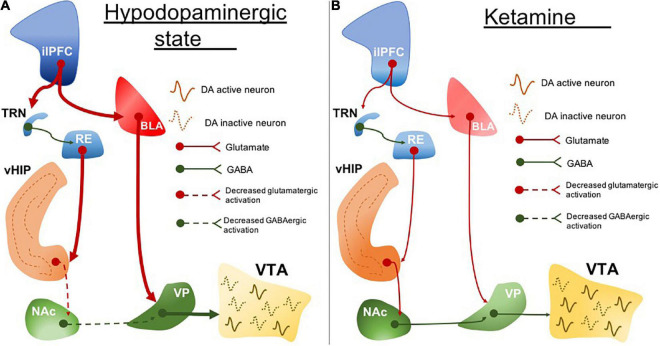
The circuit disruption underlying the hypodopaminergic state is reversed by ketamine. **(A)** ilPFC hyperactivity increases BLA activity. BLA excitatory drive induces RE hyperexcitability which also may be a resultant of ilPFC inputs through TRN. RE projections disrupt the vHIP activity and its connectivity with NAc. Impairment of vHIP activity decreases the GABAergic tone from NAc to VP. VP excitability is resulting from a decreased NAc inhibitory drive and increased BLA excitatory drive. The increased inhibitory output of VP inhibits VTA and leads to a hypodopaminergic state. **(B)** Ketamine normalizes the hypodopaminergic state by downregulating the BLA activity in response to ilPFC activity restoration. The inputs coming from BLA and ilPFC are proposed to regulate the RE activity normalization. RE may represent one important neurobiological pathway underlying the reestablishment of the vHIP and NAc connectivity. Stabilization of NAc and BLA activity decreases the activity of VP inhibitory inputs to the VTA which ultimately restores dopaminergic activity. ilPFC, the infralimbic portion of the medial prefrontal cortex; BLA, basolateral amygdala; TRN, thalamic reticular nucleus; RE, reuniens thalamic nucleus; vHIP, ventral hippocampus; NAc, nucleus accumbens; VP, ventral pallidum; VTA, ventral tegmental area. Dotted lines, decreased pathway drive.

ilPFC activity is associated with BLA excitability in rodents (Rosenkranz and Grace, 2002). Increased amygdala activity is another marked neurobiological feature in clinical depression (Drevets et al., 2008a; Yang et al., 2010; Murray et al., 2011) and it correlates with symptom severity (Mingtian et al., 2012). We found that the hypodopaminergic state caused by ilPFC activation is prevented by BLA inactivation (Patton et al., 2013). It may suggest a common circuit dysfunction feature involving the sgACC (in humans; or the ilPFC in rodents) and amygdala hyperexcitability (Figure 1A). This is important especially because these areas are pivotal in regulating stress responses. While the ilPFC drives the upregulation of BLA activity in response to stress, the plPFC has been associated with a decreased stress response by downregulating the BLA excitability (Rosenkranz and Grace, 2001, 2002). These have been associated with the opposite roles of the plPFC and ilPFC in modulating stress responses, reducing and facilitating, respectively (Lacroix et al., 2000; Radley et al., 2006; Tavares et al., 2009).

The dysfunction of the plPFC, which is analogous to the dorsal anterior cingulate cortex (dACC) in humans (Laubach et al., 2018), is also a feature described in animal models for depression. dACC disruption has been described in the human condition (Pizzagalli and Roberts, 2022) which may suggest that the inability to regulate stress response can lead to neuropathological conditions. We found that disrupting the ability of the plPFC to regulate stress during peripuberty cause animals to be more susceptible to helplessness and a hypodopaminergic state during adulthood (Uliana et al., 2020). It shows the importance of stress regulation during neurodevelopment in which unregulated stress response may lead to adult neuropathological conditions.

## Importance of reverse translation approach in animal models: Neuroimaging and epidemiological findings as valuable resources

Compared to behavioral correlates for psychiatric disorders, neurobiological alterations directly observed in patients appear to be more objectively ascertainable in research animals. These endpoints have been increasingly employed in preclinical research to complement behavioral measures. With the technological advancement in human genomics, proteomics, metabolomics, and neuroimaging, our abilities to use non-invasive approaches to assess putative biomarkers have been markedly improved. Thus, it is imperative to validate existing models and develop novel models based on “reverse translation” of clinical findings to achieve optimal model construct validity. Notably, according to the NIMH’s *Research Domain Criteria* (RDoC) initiative, one approach would be to focus animal research on neurobiological substrates relevant to normal brain functions, while being agnostic to categorical disease states. A key assumption of the RDoC framework is that psychiatric disorders arise from the deviation of essential brain functions that are defined as “domains” and “constructs,” which are controlled by neurobiological subsystems such as circuits, molecules, and genes. Thus, one benefit of the RDoC initiative is the potential to identify transdiagnostic brain dysfunctions and abnormal cognitive constructs, which could lead to insights fundamental to the development of mechanism-based targeted interventions. In addition to the emphasis on functional dimensions rather than compound disease states, the RDoC framework also explicitly advocate two equally important elements, development and environment, with circuit impact of particular environmental stressors being a central research question (Insel et al., 2010). Consistent with the RDoC framework, we argue that adolescent developmental stress may represent an effective “shared modeling strategy” to study the dopamine circuit disruptions involved in depression and schizophrenia (Grace, 2016). However, overly emphasizing transdiagnostic circuit dysfunctions could have a drawback, as this “top-down” approach could fail in its ability to understand a specifically perturbed system or rare etiological factors unique to certain disorders (Ross and Margolis, 2019) or disorders that impact multiple interacting systems, in which studying each in isolation would miss important data relevant to the disease state. This is where well-validated animal models specifically designed to study certain disorders are valuable.

The concept of “reverse translation” can be applied at various levels in preclinical psychiatry research, but traditionally it is mainly implemented *via* behavioral and/or functional characterization of risk genes. While this bottom-up approach has been undeniably useful for certain disorders with a relatively clear genetic basis, for psychiatric disorders that involve complex genetic liability and potential environmental modulation (e.g., schizophrenia and depression), it could be infeasible to comprehensively model their vast and often conflicting genetic basis (Henriksen et al., 2017). Indeed, in schizophrenia more than 200 risk loci have been identified by genome-wide association studies, which in theory could implicate even more risk genes. Moreover, schizophrenia disease risk is contributed by many common genetic variants each with a minor effect (Trubetskoy et al., 2022). Thus, at the current knowledge state, it has been argued that phenotypical impacts of common variants are difficult to model and interpret (McCarthy et al., 2014).

On the other hand, reverse translation from system-level human findings and epidemiological associations represents promising alternative strategies. One example of such “bedside-to-bench” translation is the use of neuroimaging findings to test animal models of dopamine dysregulations in schizophrenia. Two most replicated and convergent neuroimaging findings in schizophrenia are elevated striatal presynaptic dopamine function (McCutcheon et al., 2019) and structural and functional alterations in the hippocampus and the surrounding medial temporal lobes (MTL) (Lieberman et al., 2018). Although robust, these human findings are from independent studies, and whether and how these alterations are mechanistically linked to disease pathogenesis are unclear. Therefore, animal models reproducing these pathophysiological features are necessary to test for a causal relationship and/or other interactions. For example, as described previously, rodents prenatally exposed to MAM represent a promising research tool as it integrates those human findings (Modinos et al., 2015). In particular, the abnormal behavioral phenotypes of the MAM model are developed through extended trajectories consistent with the natural history of schizophrenia, with alterations related to positive symptoms that tend to manifest later compared to social withdrawal and cognitive deficits (Gomes et al., 2016). In addition, *in utero* MAM exposure is known to induce widespread, long-lasting DNA (Perez et al., 2016) and histone hypermethylation (Maćkowiak et al., 2014), which are factors supported by recent epigenetics research to be potential molecular links between genetic and environmental risk factors (Magwai et al., 2021). This gives the MAM model additional construct validity and valuable research potential, such as to identify hypermethylated genes that contribute to distinct behavioral or circuit alterations [example studies, see: Gulchina et al. (2017)]. Other reverse translation processes in schizophrenia research have been applied to generate dopamine D2 receptor overexpression and NVHL models, and their corresponding human neuroimaging findings have been reviewed extensively elsewhere (Tseng et al., 2009; Abi-Dargham, 2020). Discoveries from these models will not only causally confirm important neuroimaging findings, but also generate important testable hypotheses back to clinical research.

In addition to neuroimaging findings, epidemiological associations also represent a useful resource for reverse translation. For example, in schizophrenia, there is now compelling evidence indicating positive associations between adult psychosis risks and early exposure to diverse environmental risks (Millan et al., 2016), including age-dependent exposure to psychosocial stress, immune activation, and nutritional deficiency (Robinson and Bergen, 2021). Informed by these findings, the “epidemiology-based models” have crucial importance to advance preclinical research (Meyer and Feldon, 2010). Unlike the neuroimaging-informed models discussed above, these models tend to be based on experimental manipulations that closely mimic human environmental exposure, aiming to directly test causality implicated by human disease risk associations. These models are providing novel etiological and therapeutic insights for schizophrenia, especially on the neuroimmunological and neuroendocrine mechanisms. Furthermore, schizophrenia-associated gene and environment interaction is also being illuminated by these models, making them uniquely useful research tools to study the ontogeny of altered brain functions and possible early interventions (Meyer and Feldon, 2010).

In summary, the reverse translation of human findings into model animals represents an indispensable approach to determining if an altered neural system or environmental factor is causally linked to specific diseases. Although the RDoC framework has detailed “constructs” and units of analysis aiming to identify transdiagnostic biomarkers, it does not necessarily preclude the use of existing animal models initially designed to study a particular disease state. The key is to re-evaluate an animal model’s construct validity based on its ability to reflect the current understanding of the etiology of human disorders. In this aspect, non-invasive neuroimaging and epidemiologic risk associations have emerged to be useful resources, as they generate high-throughput data directly from human observations to update etiological theories and pathophysiological features of psychiatric disorders. These are especially important for complex disorders such as schizophrenia, for which at present there are no characterizing biomarkers and genetic phenotypes. Thus, for schizophrenia, if a model addresses a collection of consistent changes as observed in humans, ranging from risk exposure to circuit disruption and behavioral alterations, there is a higher probability that the model is of greater construct validity.

## Integrating disease risk factors for the study of schizophrenia and depression

### Stress as a major risk factor

Stress is a common environmental risk factor that can predispose individuals to various major psychiatric disorders, including depression and schizophrenia (Lupien et al., 2009; Kahn et al., 2015; Otte et al., 2016). History of adverse events across the lifespan was present in patients with depression (Richter-Levin and Xu, 2018; LeMoult et al., 2020). Indeed, stress experienced during early phases of development, such as neonatal and childhood period, also is associated with the emergence of depression later in life which may be due to the impact on the neurodevelopment trajectories of several brain areas involved with stress regulation (Liu, 2017; LeMoult et al., 2020). In animal research, stress-based models either during adulthood or early in life are widely used to approximate behavioral features of depression, such as anhedonia, amotivation, and helplessness (Pizzagalli and Roberts, 2022). These models also recapitulate core circuit dysfunction associated with depression, such as down-regulation of the dopaminergic system activity (Belujon and Grace, 2017; Kaufling, 2019). This common alteration in rodents and humans is proposed to drive depression-related behaviors (Reddy et al., 1992; Lambert et al., 2000; Nestler and Carlezon, 2006).

Schizophrenia is a neurodevelopmental disorder that usually manifests in late adolescence/early adulthood (Kahn et al., 2015; Meyer and Lee, 2019). Indeed, early life stress is a common experience in individuals who later transition to psychosis (Holtzman et al., 2012; Croft et al., 2019; Popovic et al., 2019). Different from the stress schedule in the abovementioned models to study depression, stress during neurodevelopmental periods associated with cortical maturation, such as adolescence, can lead to VTA dopamine system hyperfunction and behaviors relevant to schizophrenia (Gomes et al., 2019), which corresponds to the clinical association between childhood trauma history and adult risks for psychosis conversion (Popovic et al., 2019). Furthermore, in the MAM neurodevelopmental model of schizophrenia, it is proposed that the rats are more susceptible to stress when it is experienced during peripuberty (Zimmerman et al., 2013; Du and Grace, 2016), and their transition to adult hyperdopaminergic states can be prevented by alleviating the stress responses with an anxiolytic agent (Du and Grace, 2013) or environmental enrichment (Zhu and Grace, 2021). Drawing from these data, we postulated that sufficiently strong stressors in this prepubertal period could lead to the emergence of pathology in normal rats. This was indeed the case: exposing normal rats during this prepubertal state to combined stressors (daily footshock and three sessions of restraint stress over a 10-day period) could indeed lead to parvalbumin neuron loss, hippocampal hyperactivity, a hyperdopaminergic state, and cognitive deficits in these rats as adults (Gomes and Grace, 2017; Gomes et al., 2020). It demonstrates that the neurodevelopmental timing in which the adversity occurs may be determinant to the neuropathology, as stress is also a factor for other psychiatric conditions, with stress during the neonatal or adult period being related to depression emergence (Kendler et al., 1999; Andersen, 2015; Herbison et al., 2017; Silva et al., 2021). These convergent preclinical datasets have generated insights into the neurobiological origins of dopamine dysregulation in schizophrenia and the developmental interaction between environmental risk factors and antecedent predisposition.

**FIGURE 2:**
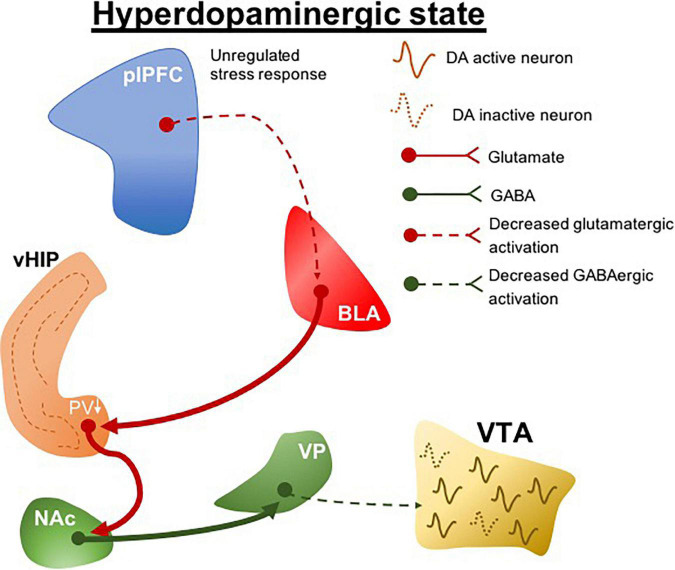
plPFC-BLA stress circuit disruption during adolescence leads to hyperdopaminergic states. Unregulated stress response by plPFC hypofunction drives increased BLA activity. The increased BLA inputs to vHIP during peripuberty underlie the reduction of PV + cells which ultimately induces a vHIP hyperactivity. Its projections activate the NAc and as a GABAergic nucleus it generates an inhibitory drive to VP; a reduction in the activity of GABAergic VP protections to the VTA produces a hyperdopaminergic state. plPFC, prelimbic portion of the medial prefrontal cortex; BLA, basolateral amygdala; vHIP, ventral hippocampus; NAc, nucleus accumbens; VP, ventral pallidum; VTA, ventral tegmental area, PV, parvalbumin. Dotted lines, decreased pathway drive.

Clinical evidence indicates that genetic risk for schizophrenia predicts structural and functional deficits in the ventromedial prefrontal cortex (Abé et al., 2021). We found that interfering with stress regulation by lesioning the prelimbic portion of the ventromedial prefrontal cortex (plPFC) rendered normal rats more susceptible to more minor stressors (Gomes and Grace, 2017). This led to the postulate that a pathology related to deficits in stress regulation could be the predisposing factor that renders an individual at risk for the development of schizophrenia (Figure 2). Indeed, what this suggests is that genetic predisposition may not in itself lead to schizophrenia; instead the predisposition may lead to an inadequate regulation of stress either through prefrontal cortical disruption or other means, which, when combined with early stress, can lead to the emergence of schizophrenia (Gomes et al., 2019). This is better correlated with the human condition, in which identical twins with the same genetic predisposition only show a 50% concordance for schizophrenia (Cardno and Gottesman, 2000), and at least 1/3 of patients with schizophrenia do not have a family history of psychosis (Kahn et al., 2015). Therefore, animal models have been instrumental in linking genetic predisposition with life stressors as etiological factors in schizophrenia. The interaction between these factors may underlie the unregulated stress responses that precipitate pathological conditions, which potentially drive individual differences in the susceptibility to stress-related disorders. Although these variables have long been suggested in clinical association studies to modulate disease risks, how they contribute to the pathophysiological processes of diseases is unclear. Clinical research assessing stress-related risk factors for psychiatric conditions tends to be retrospective in nature, so that it typically precludes detailed segregation of variables, for example, the stressor type and intensity. However, these stress-specific characteristics could distinctively precipitate a particular neuropathological condition.

Preclinical studies have significantly advanced our understanding of how stress-specific variables influence the neurobiological/circuit mechanisms underlying complex behaviors involved in depression and schizophrenia (Lipska and Weinberger, 2000; Meyer and Feldon, 2010; Czéh et al., 2016; Muir et al., 2018). These animal models permit studies of potential prevention and therapeutic interventions, along with the ability to investigate early biomarkers. Although each single animal model certainly cannot recapitulate all the aspects of complex human disorders, they can bring unique and often unexpected insights that are the building blocks for novel therapies. Also, the clinical behavioral and neurobiological circuit dysfunction differ on the individual level (Otte et al., 2016; Su and Si, 2022), which reflects that even human conditions are not homogeneous. Therefore, regarding protocols used to understand some aspects of behavioral and neurobiological features of depression and schizophrenia, stress seems to be a mutual variable in both rodent models and the human condition.

The effect of stress can change over time, and the outcomes depend on when the stress is presented. For example, repeated stress acutely increases VTA dopamine system activity (Valenti et al., 2011b). However, repeated stress causes rats to show a hypodopaminergic state 1 week after the end of stress (Chang and Grace, 2014; Gomes et al., 2020). This switch in the dopaminergic state is likely due to cumulative neurobiological disruptions in the brain areas involved in stress regulation. The acute effect of stress is dependent on ventral hippocampus (vHip) activation (Valenti et al., 2011b) which drives the hyperdopaminergic state by activating the nucleus accumbens (NAc) and disinhibition of ventral pallidum GABAergic projection to the VTA (Grace, 2016). On the other hand, as mentioned above, the increased activity in the ilPFC underlies the hypodopaminergic state in rats subjected to CMS (Moreines et al., 2017b). The ilPFC excitability observed in animal models of affective dysregulation may disrupt the vHip activity indirectly *via* the reuniens thalamic nucleus (Dolleman-van der Weel and Witter, 2020), which also has been shown to regulate amotivational behaviors and dopaminergic activity (Zimmerman and Grace, 2016; Kafetzopoulos et al., 2018). Rats expressing helplessness have a disruption of long-term potentiation (LTP) in vHip-NAc plasticity (Belujon and Grace, 2014). In normal rats, vHip high-frequency stimulation induces LTP in NAc which is switched to long-term depression in helplessness (Belujon and Grace, 2014). It reflects abnormal vHip modulation of synaptic plasticity in the NAc. It can ultimately contribute to the decreased reward-related medial VTA dopamine system activity observed in helplessness rats (Belujon and Grace, 2014, 2017). All the circuit dysfunction information above takes advantage of animal models based on stress exposure to study changes related to depression, which cannot be observed in naïve animals.

Early life stress, such as childhood trauma, is associated with an increased risk for schizophrenia and depression later in life (Redman et al., 2017; Croft et al., 2019; Devi et al., 2019; Popovic et al., 2019; LeMoult et al., 2020). Stress early in life can be particularly detrimental because individuals are still undergoing neurodevelopmental changes (Bouwmeester et al., 2002; Marco et al., 2011; Gee et al., 2013b; Spear, 2013; Casey et al., 2019), and it can profoundly affect brain area maturation and connectivity (Bath et al., 2016; Johnson et al., 2018; Herzberg and Gunnar, 2020). One area implicated in the pathophysiology of depression and schizophrenia is the PFC (Perlstein et al., 2001; Rogers et al., 2004; Drevets et al., 2008a; Selemon and Zecevic, 2015), which is the last region to reach maturation during early adulthood (Gogtay et al., 2004; Davey et al., 2008). Dysfunctional activity in the PFC areas is observed in patients with depression and schizophrenia with a history of childhood adversity (Kim et al., 2019; Popovic et al., 2019; González-Acosta et al., 2020; Silva et al., 2021), which may be a long-term consequence of unregulated stress response. In the context that PFC is not adequately developed to regulate stress, it can allow the upregulation of amygdala activity early in life and affect their connectivity. In particular, early stress appears to induce precocious development of BLA-plPFC connectivity. Indeed, we have shown in rats that peripubertal stress induces precocious development of plasticity in the BLA projections to the plPFC (Uliana et al., 2021). This is consistent with clinical data showing accelerated connectivity between the amygdala and PFC in individuals with a history of maternal deprivation (Gee et al., 2013a). This early abnormal connectivity would interfere with the normal development of stress coping responses, which enables early coping skills at the cost of inefficient stress regulation in adulthood (Callaghan and Tottenham, 2016; Herzberg and Gunnar, 2020). Therefore, it may represent a common circuit dysregulation in rodents and humans after early life adversities (Gee et al., 2013a; Bath et al., 2016; Callaghan and Tottenham, 2016), which highlights the importance of using animal models to elucidate neurobiological features in the human conditions.

### Genetic contribution to stress susceptibility

Schizophrenia and depression tend to run in the same families (Hovens et al., 2010), which suggests a common genetic link (Cross-Disorder Group of the Psychiatric Genomics Consortium, 2013). Depression and schizophrenia share several genetic variations associated with these psychiatric disorders (Brainstorm Consortium et al., 2018). Individuals at ultra-high risk for schizophrenia that do not transition to psychosis show an increased incidence of affective disorders and depression in adulthood (Lin et al., 2015; Chan et al., 2018). The differences in susceptibility may be a product of interaction between genetic and environmental factors during a specific neurodevelopmental period (Grace, 2016; Meyer and Lee, 2019). Stress and its regulation may have a key role in this divergence, particularly depending on the developmental period (Green et al., 2010; McLaughlin et al., 2010; Andersen, 2015; Brenhouse and Bath, 2019). For example, the same repeated stress protocol induces a hyperdopaminergic schizophrenia-like state in adulthood when applied to adolescent rats and a hypodopaminergic depression-like state when applied to adult rats (Gomes et al., 2020). This reflects that both states may share the same risk factor with the age of exposure being a determinant of the outcome. Indeed, factors that impact stress regulation across the lifespan are essential for the neuropathological consequences (Lupien et al., 2009; McEwen, 2017; Shaw et al., 2020). The interference with plPFC regulation of stress during the peripubertal period leads to increased susceptibility to helplessness and hypodopaminergic state in adult rats (Uliana et al., 2020). However, the plPFC disruption during the same period when combined with mild stressor exposure leads to an adult hyperdopaminergic state (Gomes and Grace, 2017). The impairment in stress regulation may represent a preexisting vulnerability at the individual level in which the environmental factors would be determinant for the neuropathological state later in life. Alternately, if the early life trauma is sufficiently extreme, this can lead to adult pathology even without a family history (Devi et al., 2019; Popovic et al., 2019). We posit that the common developmental factor that underlies risk for multiple psychiatric disorders is either extreme peripubertal stress in normal individuals (e.g., severe childhood trauma) or developmentally driven peripubertal hyper-responsivity to mild stressors (e.g., stressful life events) in those at genetic risk or with other predispositions. Thus, adult stress resilience/susceptibility has been proposed to be attained through a developmental process (Nederhof and Schmidt, 2012), which can be critically shaped by early experience, brain development, and maturation (Andersen, 2015; Peña et al., 2019).

The preexistent genetic vulnerability to major psychiatric conditions may determine the impact of environmental influence during neurodevelopment. Animal models to study disease states are essential to study each factor’s contribution and their interaction in the disease pathogenesis. Although the animal models for depression and schizophrenia are important for new therapeutic discoveries, preventive interventions and early markers represent a promising and necessary field of study. The early detection of markers for specific neuropsychiatric conditions can inform the clinician of the timing for preventive measures, including targeting disease transition prevention or alleviating the severity of a future disease state. Therefore, animal models are a crucial tool to evaluate these aspects for better clinic and preclinic crosstalk.

## Not our fault! Animal models should not be blamed for failures in drug development

While the back-and-forth translation of animal models and clinical studies has demonstrated value in advancing our understanding of disease states, there has been a move advanced by the US National Institute of Mental Health (NIMH) and others that eschews the use of animal models of human diseases. A substantial amount of this is due to the failure of animal models to provide novel targets for pharmacological interventions that are effective in schizophrenia. Thus, animal models have advanced a circuit in which loss of hippocampal parvalbumin interneurons leads to hippocampal hyperactivity and, *via* the striatum and ventral pallidum, disinhibition of the dopamine system and a resultant hyperdopaminergic state (Grace, 2016; Sonnenschein et al., 2020). This circuit is supported by strong clinical evidence, showing parvalbumin neuron loss in the hippocampus (Zhang and Reynolds, 2002; Konradi et al., 2011) and hippocampal hyperactivity (Schobel et al., 2013) that occurs in concert with increased dopamine function (Grace, 2012; Modinos et al., 2021). However, when pharmaceutical companies have targeted this circuit, the resultant agents have failed to demonstrate efficacy despite promising results from early trials. Thus, Lilly developed a metabotropic glutamate receptor 2/3 (mGluR2/3) agonist to attenuate glutamate hyperfunction (Rorick-Kehn et al., 2007); Roche developed a glycine uptake inhibitor to potentiate NMDA receptor drive presumably of the remaining parvalbumin interneurons (Alberati et al., 2012), and Pfizer developed a phosphodiesterase type 10 (PDE10) inhibitor to attenuate hippocampal overdrive of the striatum (Menniti et al., 2020). While each of these agents showed promise in animal models and early phase trials, each failed in the large multi-center phase three trials. The consequence is that many in industry and the NIMH concluded that animal models are not good predictors of the therapeutic efficacy of drugs. However, our studies have shown such a conclusion to be short-sighted; i.e., it is not the animal models that are failing, but instead a reliance on a pharmaceutical trial model that has been effective at identifying new dopaminergic modulatory drugs, but in all likelihood will fail to identify an effective drug acting on a novel target. This is due to the trial design—i.e., in these large trials, the companies typically rely heavily on chronic patients with schizophrenia that have been treated previously for many years with standard D2 antagonist drugs. As per protocol, the patients are withdrawn from their current medications for 1–2 weeks to wash out residual drug, before the novel agent is tested. However, this is problematic—treatment with a D2 antagonist will induce DA supersensitivity due to the system increasing in the number of D2 receptors to compensate for the blockade (Servonnet and Samaha, 2020). However, when these receptors are uncovered by washout of the drug, there remains a strong dopamine supersensitivity. As a result, when withdrawing a D2 antagonist, the only agent that can restore efficacy is another D2 antagonist. Indeed, we found that if we treated a MAM rat with the antipsychotic agent haloperidol for just 3 weeks and withdrew the drug for 1 week, a novel agent that was highly effective in the untreated MAM rat was now without efficacy (Gill et al., 2014)–i.e., by treating with haloperidol, we replaced hippocampal driven dopamine hyperactivity with a postsynaptic D2 supersensitivity. As a result, we believe that several potentially highly effective agents were prematurely dismissed not because the animal models were ineffective, but because the clinical trial design was not effective at evaluating agents with novel targets. Indeed, our studies have suggested that the mGluR2/3 agonist pomaglumetad should be a highly effective antipsychotic agent with minimal or no D2 antagonism-related side effects (Sonnenschein and Grace, 2020). Interestingly, although pomaglumetad failed to show efficacy in phase 3 trials, later analyses indicated this drug was effective in some patients, particularly those who were in the earlier disease stages (Kinon et al., 2015). These findings indicate that the efficacy of pomaglumetad in schizophrenia may depend on treatment history and disease progression.

One important issue is the necessity to use animal models of disorders to study the effectiveness of novel compounds. This is because a drug that targets a deficit state may not produce measurable results in a normal system, or the results may be altered in nature. One example would be the use of antipsychotic drugs. Administering antipsychotic drugs to normal rats has provided some insights into mechanisms, such as the importance of D2 blockade in their therapeutic action (Carlsson and Lindqvist, 1963) and the correlation between D2 affinity and clinical potency (Seeman et al., 1976). However, the drugs are likely not working the same in normal animals. For example, we had shown that it requires 3–4 weeks of treatment with first- or second-generation antipsychotic drugs to induce dopamine neuron depolarization block (Bunney and Grace, 1978; Chiodo and Bunney, 1983), which is proposed to be the therapeutic action *via* decreasing the pathologically high population activity of the dopamine system (Grace and Gomes, 2019). However, this is not consistent with the clinical picture, in which antipsychotic drugs can exert clinical efficacy within days of administration (Agid et al., 2006). When antipsychotic drugs are first administered to rats, the first effect is to increase dopamine neuron population activity (i.e., the number of neurons firing). However, in the MAM model, there is already an increased population activity at baseline (Lodge and Grace, 2007); now when the antipsychotic drug is administered to the MAM rat, it acts upon an already up-regulated system to rapidly induce depolarization block (Valenti et al., 2011a), unlike that observed in the control animal. The same is likely true for the rapid and sustained actions of the novel antidepressant ketamine. In our learned helplessness depression model in rats, ketamine rapidly reverses dopamine neuron down-regulation and hippocampal-accumbens long-term depression (LTD); an effect that is not observed in naïve animals (Belujon and Grace, 2014; proposed circuit in Figure 1B). In addition, the potential antidepressant effects of quetiapine on the dopamine system were again only observed in an animal model for depression; no consistent similar effect is seen in naïve animals (Moreines et al., 2017a). Therefore, while drug administration to naïve animals may provide some insight into targets and mechanism of specific behavior and circuit, administering it to a well-developed animal model is essential to provide insights into neurobiological mechanism leading to its therapeutic action in a pathological context.

## Conclusion

In summary, whereas psychiatric disorders are traditionally modeled *via* acute pharmacological manipulation of neurotransmission and behavioral characterization, increasing animal models are now based on reverse translation of genetic association, neuroimaging, and epidemiological findings. By complementing behavioral readouts with clinically relevant findings, animal models based on diverse reverse translation strategies can have great construct validity. Notably, no model is “perfect” nor sufficient to capture all aspects of psychopathology (Lipska and Weinberger, 2000). Thus, it is important to emphasize clinically relevant dysfunctions, use multidisciplinary approaches, and rely on collaborative efforts to tackle complex disorders.

## Author contributions

DU constructed the figures. All authors were involved in the concept, writing, citations, and finalization of the document and approved the submitted version.
